# Top 10 Public Health Challenges in 2025: Lessons and Priorities for 2025–2030

**DOI:** 10.1002/puh2.70372

**Published:** 2026-09-15

**Authors:** Don Eliseo Lucero Prisno, Mohamed Mustaf Ahmed, Zhinya Kawa Othman, Rahmatullah Nazari, Uthman Okikiola Adebayo, Olalekan John Okesanya, Shuaibu Saidu Musa, Francesco Branda, Abdullahi Hassan Elmi, Jerico Bautista Ogaya

**Affiliations:** ^1^ Department of Global Health and Development London School of Hygiene and Tropical Medicine London UK; ^2^ Faculty of Medicine and Health Sciences SIMAD University Mogadishu Somalia; ^3^ Faculty of Pharmaceutical Sciences Chulalongkorn University Bangkok Thailand; ^4^ Faculty of Dentistry Cheragh Medical University Kabul Afghanistan; ^5^ Department of Digital Health Global Health Focus Africa Kigali Rwanda; ^6^ Department of Medical Laboratory Science Neuropsychiatric Hospital, Aro Abeokuta Nigeria; ^7^ School of Global Health Faculty of Medicine Chulalongkorn University Bangkok Thailand; ^8^ Unit of Medical Statistics and Molecular Epidemiology Campus Bio‐Medico University of Rome Rome Italy; ^9^ Department of Nursing Faculty of Medicine and Health Sciences Dr. Sumait Hospital, SIMAD University Mogadishu Somalia; ^10^ Department of Medical Technology Institute of Health Sciences and Nursing Far Eastern University Manila Philippines

**Keywords:** climate and health, global health, health equity, health systems strengthening, pandemic preparedness, public health challenges

## Abstract

By 2025, global public health will be shaped by interconnected and intensifying threats that demand coordinated international action. Ten key public health challenges were identified and examined. This perspective reviews the challenges that shaped 2025 and the priorities set for the period 2025–2030, using evidence available up to July 2026; therefore, it is in part a retrospective assessment of that year rather than solely a prospective identification of challenges at its outset. The designation of the Top 10 denotes prominence and salience rather than a strict ordinal ranking. The challenges selected for this year include drug‐resistant infections, health consequences of a warming planet, rising rates of psychological distress, persistent burden of long‐term diseases and health inequities, preparedness for future disease outbreaks, widespread hunger and poor nutrition, shortages of doctors and nurses, harmful use of alcohol and drugs, barriers to reproductive and sexual well‐being, and ethical questions raised by the growing role of computing and data‐driven tools in medicine. Each challenge was examined in terms of its current scope, the populations most affected, and the strategies needed to mount an effective response to it. The analysis draws out the cross‐cutting links among the 10 challenges and sets out a proposed agenda for action for 2025–2030. The adoption of the World Health Organization Pandemic Agreement by the World Health Assembly in May 2025 established an important framework for future cooperation, although the agreement had not been opened for signature and had not entered into force at the time of writing, and progress across most indicators continued to fall short of global targets. Pressures that intensified in 2025, notably the retrenchment of international health financing and the rapid clinical adoption of artificial intelligence ahead of rigorous evaluation, further sharpened several of these challenges.

## 1 Introduction

Global public health is shaped by the convergence of persistent and emerging threats that demand coordinated, evidence‐based responses. The aftermath of the COVID‐19 pandemic continues to reverberate across health systems worldwide, exposing the fragility of disease surveillance, emergency response capacity and equitable access to essential services [1]. As the World Health Organization (WHO) has warned, global health gains are slowing, with progress on key indicators stalling or reversing in several regions [2]. Noncommunicable diseases (NCDs) continue to account for approximately 75% of all non‐pandemic‐related global deaths [3], whereas infectious disease outbreaks, antimicrobial resistance (AMR), and climate‐related health emergencies are intensifying [4]. More than one billion people are estimated to live with mental health conditions globally [5]. Meanwhile, an estimated 4.6 billion people were not fully covered by essential health services in 2023, and on the separate financial protection dimension of universal health coverage, 2.1 billion people faced financial hardship from out‐of‐pocket health spending in 2022, including 1.6 billion living in poverty or pushed deeper into it [6, 7]. The service coverage index rose from 54 to 71 out of 100 between 2000 and 2023, although progress has slowed over the past dfdeade [8].

This perspective identifies and examines 10 key public health challenges in 2025, building on earlier assessments of global health priorities [1, 9]. The selection of challenges was guided by their global relevance, degree of urgency, and scope for impactful public health interventions [1]. The 10 challenges are as follows: AMR, climate change and health, mental health burden and treatment gaps, NCDs and health disparities, pandemic preparedness and infectious disease threats, food insecurity and malnutrition, health workforce shortages, substance use and substance use disorders, sexual and reproductive health equity, and artificial intelligence (AI), digital health and public health ethics. These challenges highlight shared concerns that require proactive, collaborative and innovative responses to strengthen the resilience of public health systems worldwide and promote community well‐being on a global scale. Table 1 summarizes the rationale for including each of these 10 challenges.

**TABLE 1:** Top 10 public health challenges in 2025 and their rationales.

Public health challenge	Rationale for inclusion in 2025	Reference(s)
Antimicrobial resistance	1 in 6 laboratory‐confirmed infections are resistant (2023); 1.27 million deaths are attributable (2019); 1.91 million deaths are projected by 2050; and up to $3.4 trillion in annual losses to gross domestic product is projected by 2030	[10, 11, 12]
Climate change and health	Twelve of 20 Lancet Countdown indicators at record levels (2025); 546,000 heat‐related deaths a year (modelled); 640 billion labour hours and $1.09 trillion lost (2024); burden concentrated in Africa and Asia	[13]
Mental health burden and treatment gaps	More than 1 billion people are affected (2021, modelled); 727,000 suicides (2021); depression and anxiety cost $1 trillion a year (modelled); service coverage is 8% in low‐income countries; and the median budget share is 2%	[5, 14]
Noncommunicable diseases and health disparities	In 2021, there were 43 million deaths, 75% of non‐pandemic mortality (), and 82% of premature NCD deaths in LMICs (), and the 2011 United Nations target of a 25% reduction by 2025 was largely missed, with only 1.5% progress in a decade	[3, 15]
Pandemic preparedness and infectious disease threats	The WHO Pandemic Agreement was adopted on 20 May 2025, but is, 2025, not yet open for signature or in force. Over 14 million infants are unvaccinated, and measles is re‐emerging. WHO priority pathogens require sustained research and development	[16, 17]
Food insecurity and malnutrition	673 million people are affected by hunger; and 2.3 billion are food insecure (2024); 2.6 billion are unable to afford a healthy diet (2024); and hunger is still rising in Africa, above 20% (2024)	[18]
Health workforce shortages	A shortfall of 11 million health workers is projected by 2030, concentrated in low‐ and lower‐middle‐income countries; workforce capacity constrains every other priority	[19, 20]
Substance use and substance use disorders	In 2023, 316 million people used drugs (2023), an increase of 28% in a decade; 2.6 million alcohol‐attributable deaths occurred annually (modelled); 1 in 12 individuals with drug use disorders were treated (2023); and youth‐targeted nicotine products threaten tobacco‐control gains	[21, 22, 23]
Sexual and reproductive health equity	260,000 maternal deaths (2023), 164 million women with an unmet need for contraception, and intimate partner or sexual violence affected 1 in 3 women in their lifetime, with approximately 21 million adolescent pregnancies per year in LMICs (modelled)	[24, 25, 26]
Artificial intelligence, digital health and public health ethics	Rapid clinical adoption raises ethical, equity, regulatory and data protection questions; the WHO AI ethics guidance was updated for large multimodal models (2024); GI‐AI4H was launched to harmonize governance; and the digital divide persists in LMICs	[27, 28]

*Note:* ‘Top 10’ denotes prominence rather than rank; challenges are listed thematically. Years in parentheses are the data year of an observed value; modelled estimates and projections are labelled as such.

Abbreviations: GLASS, WHO Global Antimicrobial Resistance and Use Surveillance System; LMICs, low‐ and middle‐income countries.

The selection of these challenges followed a structured, consensus‐based process to improve transparency. The challenges were identified through discussions among the authors, a multidisciplinary group of public health researchers, and practitioners, rather than by independent scoring, and these criteria were applied qualitatively rather than through a formal weighting or Delphi procedure. Differences of view were resolved by continued discussion until the group agreed on the final list, and the supporting evidence was updated to July 2026 [1, 9]. The designation of Top 10 signals prominence and salience rather than a strict ordinal ranking, and the 10 challenges are presented thematically rather than in order of priority. Several other important issues were considered but were not retained as standalone challenges, including air pollution, conflict and displacement, population ageing, health financing, vaccine hesitancy, water, sanitation and hygiene, child health and the commercial determinants of health. Many of these are cross‐cutting and are addressed within the selected challenges, for example, vaccine hesitancy within pandemic preparedness, the commercial determinants of health within NCDs, water, sanitation and hygiene together with displacement within food insecurity and climate change, and health financing within the health workforce and the proposed action agenda in Section 12. This scoping reflects a deliberate focus on a tractable set of priorities amenable to coordinated global action, rather than an exhaustive enumeration of all public health concerns.

## 2 Antimicrobial Resistance

AMR has emerged as one of the most pressing threats to global public health by 2025, undermining the effectiveness of life‐saving treatments and placing populations at a heightened risk of common infections [29]. The WHO Global Antibiotic Resistance Surveillance Report 2025 found that in 2023, one in six laboratory‐confirmed bacterial infections captured by the WHO Global Antimicrobial Resistance and Use Surveillance System (GLASS) was resistant to standard antibiotics. The estimate covers eight common pathogens causing bloodstream, urinary tract, gastrointestinal, and urogenital gonorrhoea infections reported by more than 100 countries; therefore, it describes infections captured by that surveillance system rather than all bacterial infections worldwide [10]. AMR rose in more than 40% of the bacteria‐drug combinations tracked between 2018 and 2023, with average annual increases ranging from 5% to 15% [10].

Geographic disparities in resistance rates are substantial, with one in three infections in the WHO South‐East Asia and Eastern Mediterranean regions being resistant to antibiotics, compared with one in five in the African region. These comparisons should be read with caution because 48% of countries did not report to GLASS in 2023, and about half of those reporting lacked the systems to generate reliable data; thus, differences in laboratory and surveillance capacity may shape the regional estimates [10]. Bacterial AMR was directly responsible for an estimated 1.27 million deaths in 2019 and contributed to nearly 5 million deaths globally that year [12]. A peer‐reviewed forecast estimated 1.91 million deaths directly attributable to and 8.22 million deaths associated with bacterial AMR in 2050 under its reference scenario [30]. Under the modelled scenario in which resistance continues to rise, AMR could also reduce the global gross domestic product by up to $3.4 trillion per year by 2030 [11].

The 2024 United Nations General Assembly political declaration on AMR reaffirmed global commitments to tackle resistance through a One Health approach that integrates human, animal and environmental health [31]. Strengthening global surveillance systems, investing in research and development of new antimicrobials and implementing antibiotic stewardship programmes are core components of an effective response [10]. AMR does not act in isolation, and its interactions with climate change, health system disruption and One Health vulnerabilities are examined further in the cross‐cutting analysis in Section 12 [4]. In the short term, priorities include expanding WHO GLASS surveillance coverage, enforcing stewardship under the WHO AWaRe classification and curbing over‐the‐counter antibiotic sales, whereas long‐term progress depends on sustained investment in novel antimicrobials, vaccines and diagnostic and laboratory capacity in low‐ and middle‐income countries (LMICs) [10].

## 3 Climate Change and Health

Climate change continues to pose a substantial and growing threat to human health in 2025, with the 2025 Lancet Countdown report finding that 12 of the 20 key indicators tracking health threats have reached record levels [13]. The global mean surface temperature has increased substantially over recent decades, reaching the highest level on record in 2023, 1.17°C above the 1951–1980 average, compared with −0.07°C in 1951 [32]. The average person was exposed to a record 16 additional health‐threatening hot days in 2024 as a direct result of climate change, whereas infants and older adults experienced an all‐time high of 20 heatwave days per person [13]. Heat‐related deaths have risen by 23% since the 1990s, reaching an estimated 546,000 deaths per year (modelled estimate), whereas heat exposure in 2024 led to the loss of 640 billion potential labour hours and productivity losses valued at $1.09 trillion [13].

The health consequences of climate change extend beyond that of heat exposure. Droughts and heatwaves are associated with an additional 123.7 million people facing moderate or severe food insecurity in 2023 [13]. Africa, despite contributing approximately 4% of global greenhouse gas (GHG) emissions, bears a disproportionate share of the health consequences of climate change [13]. Africa emitted 0.8 Gt of energy‐related carbon dioxide (CO_2_) in 2022 [32], whereas the total global emissions of all GHGs that year were 57.4 Gt of CO_2_ equivalent (GtCO_2_e) [33]. The two quantities are not directly comparable because the first covers carbon dioxide alone, and the second aggregates all GHGs; the like‐for‐like comparison is the 4% share noted above.

The WHO has called for climate‐resilient and sustainable health systems and identified migrants and displaced populations as among those most affected by climate‐related disasters [34]. Integrating health considerations into climate policies, strengthening the surveillance of climate‐sensitive diseases and securing dedicated financing for health within climate frameworks remain priorities [34]. The pathways linking climate change to food insecurity, undernutrition and infectious disease susceptibility are further developed in the cross‐cutting analysis in Section 12 [13].

## 4 Mental Health Burden and Treatment Gaps

**FIGURE 1:**
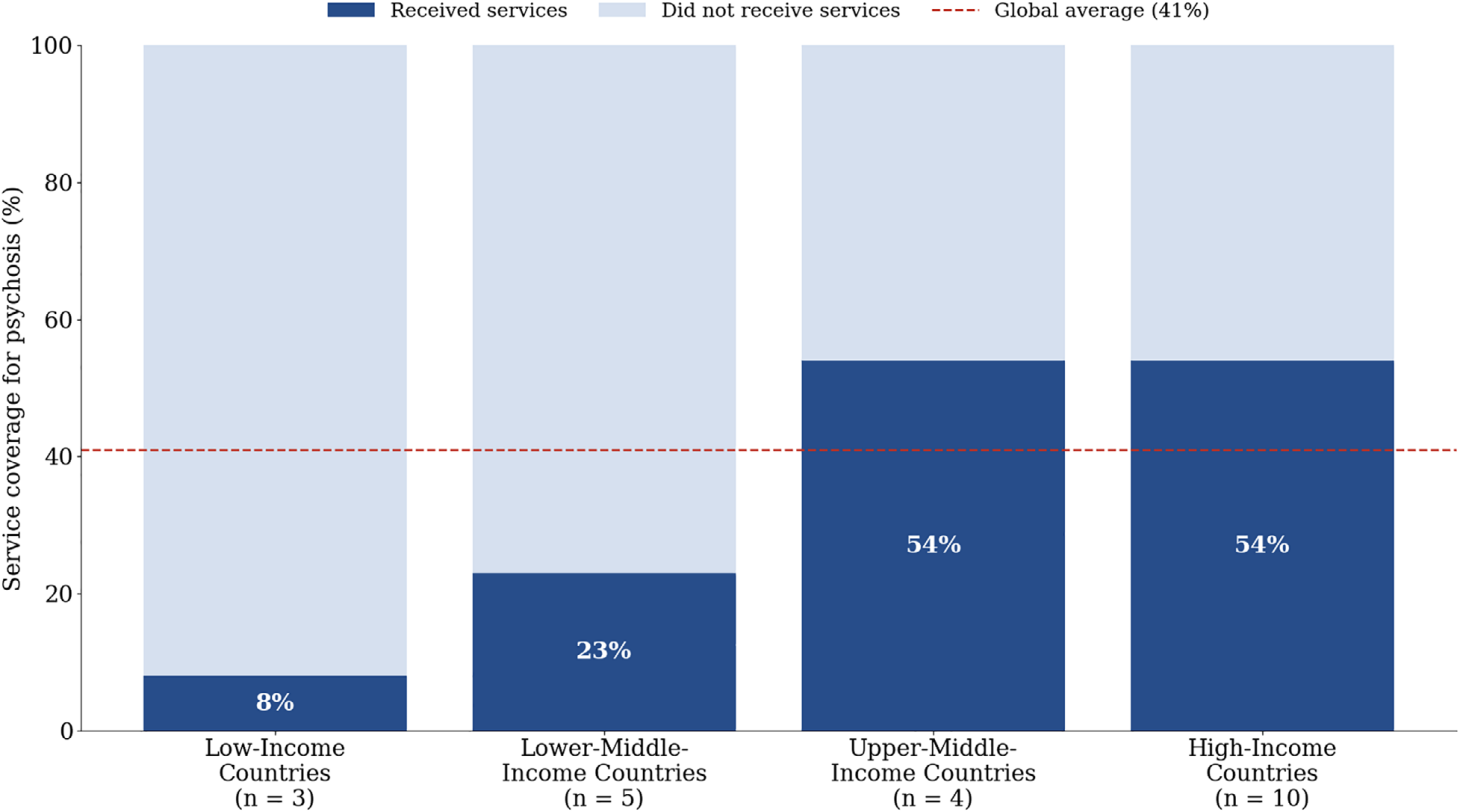
Service coverage for psychosis by the World Bank country income group. Author‐generated figure based on the World Health Organization's Mental Health Atlas 2024 [35].

More than 1 billion people are estimated to be living with a mental health condition worldwide, on 2021 prevalence estimates, and the burden falls most heavily on populations with the least access to care [5]. Mental health conditions represent the second largest reason for long‐term disability globally, and depression and anxiety alone cost the global economy an estimated $1 trillion a year in lost productivity [5], a loss that modelling of scaled‐up treatment suggests could be substantially offset [14]. An estimated 727,000 people died by suicide [5]. The treatment gap remains wide and is strongly influenced by national income. Service coverage for psychosis, the indicator WHO reports by income group, is 8% in low‐income countries, 23% in lower middle‐income countries, and 54% in both upper middle‐income and high‐income countries, against a global average of 41% [35]. These estimates are presented in Figure 1. The median government spending on mental health remains at just 2% of total health budgets, a figure unchanged since 2017, whereas disparities between countries are extreme, with high‐income countries spending up to $65 per person compared with as little as $0.04 in low‐income countries [5, 36].

The COVID‐19 pandemic was associated with an additional 53.2 million cases of major depressive disorder (an increase of 27.6%) and 76.2 million cases of anxiety disorders (an increase of 25.6%) globally in 2020 [37], and lockdowns, physical isolation and the shift to remote work and online schooling increased loneliness rates globally [1]. The WHO Commission on Social Connection, established in late 2023, identified loneliness as a public health issue; pooled cohort evidence links poor social relationships to a 29% higher risk of incident coronary heart disease and a 32% higher risk of stroke [38]. Scaling up investment in community‐based mental health services and integrating mental health into primary care are essential for closing this gap [35]. These stressors are deeply interconnected, as climate‐related displacement, food insecurity, conflict and gender‐based violence all heighten psychological distress, a syndemic pattern examined in Section 12 [39, 40]. In the near term, task‐shifting and the WHO Mental Health Gap Action Programme intervention guide can extend care where specialists are scarce [41], whereas durable progress requires raising the mental health share of health budgets above the current 2% median and embedding mental health within the universal health coverage [5].

## 5 NCDs and Health Disparities

These treatment gaps are within the wider burden of chronic diseases. NCDs, including cardiovascular disease (CVD), cancer, chronic respiratory diseases and diabetes, remain the leading cause of death globally, accounting for at least 43 million deaths in 2021, equivalent to 75% of all non‐pandemic‐related mortality [3]. CVD alone accounted for at least 19 million deaths in 2021, followed by cancers at 10 million, chronic respiratory diseases at 4 million and diabetes at over 2 million, a figure that follows the WHO source, including kidney disease deaths caused by diabetes [3]. In the WHO European Region alone, NCDs cause 1.8 million avoidable deaths annually and productivity losses estimated at over $514.5 billion [42].

The burden of NCDs falls disproportionately on LMICs, where 82% of premature deaths from these conditions occur [3]. Health inequalities persist across all social groups worldwide, irrespective of a country's level of development or income status. Populations such as refugees, migrants, Indigenous peoples, persons with disabilities, women, children, rural communities and ethnic minorities continue to encounter systemic obstacles, discrimination and restricted access to quality healthcare [43]. The 2011 United Nations target of reducing premature mortality from NCDs by 25% by 2025 has largely fallen short, with global progress improving by only 1.5% over the past decade [15].

Removing systemic barriers and achieving fair access to healthcare requires addressing these issues through the lens of social and commercial factors that shape health outcomes [15]. Expanding early diagnosis, reducing modifiable risk factors such as tobacco use, alcohol consumption, physical inactivity and poor diet, and prioritizing primary prevention strategies are essential to reversing these trends [15, 44].

## 6 Pandemic Preparedness and Infectious Disease Threats

Chronic diseases are not the only pressure on health systems, and infectious threats returned to the centre of global health governance in 2025. The adoption of the WHO Pandemic Agreement by the World Health Assembly on 20 May 2025 represented an important development in global pandemic governance; it was only the second instrument adopted under Article 19 of the WHO Constitution, after the WHO Framework Convention on Tobacco Control [17, 45]. Reached after more than 3 years of negotiations and adopted by 124 governments, the agreement focuses on equitable access to vaccines, diagnostics and treatments, as well as strengthening disease surveillance through a One Health approach [17, 46]. However, adoption is only one step in a longer legal process. The agreement will be open for signature only after an annex establishing the pathogen access and benefit‐sharing (PABS) system is negotiated by an intergovernmental working group and adopted by the World Health Assembly. It then required ratification by individual states and entered into force 30 days after the sixtieth ratification. At the time of writing, the annex remained under negotiation; therefore, the agreement had not been opened for signature, had not been ratified, had not entered into force, and its provisions were not yet operative for states [17, 46].

Emerging and re‐emerging infectious diseases continue to pose significant threats, with the WHO Research and Development Blueprint for Epidemics prioritizing research on high‐risk pathogens, including Rift Valley fever, Ebola, Marburg virus disease and other epidemic‐prone and emerging threats, such as avian influenza and Oropouche disease [47, 48]. Childhood vaccination rates in several countries are declining to levels not seen in many years, driving a marked increase in measles cases and deaths. Over 14 million infants remain unvaccinated globally, and misinformation, rather than vaccine safety or efficacy, has become one of the greatest threats to these hard‐won immunization gains [16]. Strengthening primary healthcare systems, investing in genomic surveillance infrastructure and countering vaccine misinformation through community engagement and health literacy are needed to maintain gains against infectious diseases [1, 49]. Realizing the potential of the agreement will require the completion of outstanding legal steps, together with sustained political will and dedicated resources for implementation [50].

## 7 Food Insecurity and Malnutrition

**FIGURE 2:**
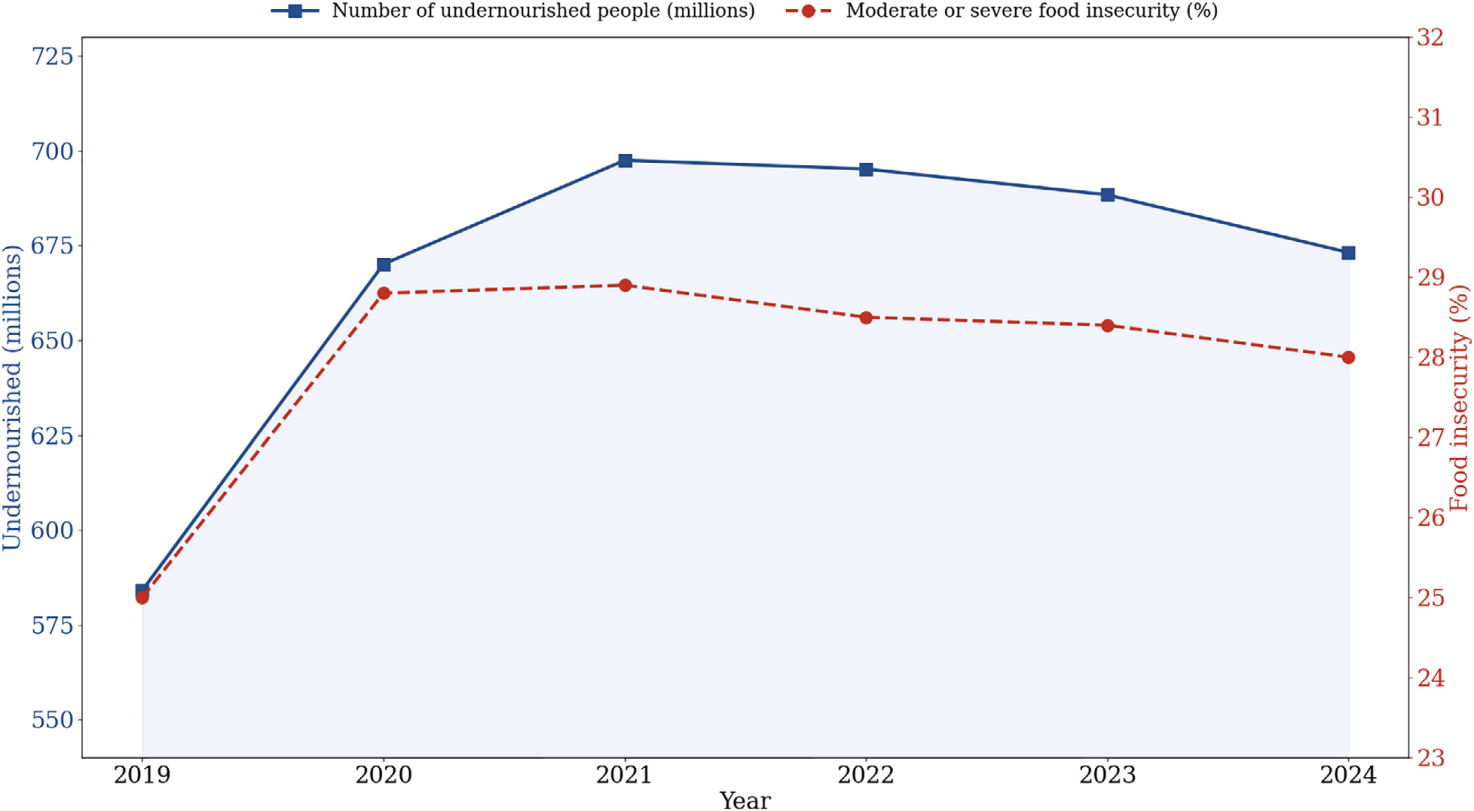
Undernourishment and moderate or severe food insecurity worldwide, 2019–2024. Author‐generated figure based on The State of Food Security and Nutrition in the World 2025 [18].

Global food insecurity and malnutrition will remain critical public health challenges in 2025. The 2025 State of Food Security and Nutrition in the World report revealed that an estimated 673 million people, or 8.2% of the global population, experienced hunger in 2024, a slight decline from 8.5% in 2023 [18]. However, approximately 2.3 billion people experienced moderate or severe food insecurity in 2024, and hunger continued to rise in most subregions of Africa and Western Asia, where, in Africa, the proportion exceeded 20%, affecting 307 million people, whereas in Western Asia, it reached 12.7%. The trend since 2019 is shown in Figure 2 [18].

Anaemia among women aged 15–49 years has increased from 27.6% to 30.7%, with most regions showing stagnation or deterioration [18]. As of 2024, 2.6 billion people still cannot afford a healthy diet, as elevated inflation in many countries has undermined purchasing power, particularly among low‐income populations [18]. In low‐income countries, the number of people who cannot afford a healthy diet increased from 464 million in 2019 to 545 million in 2024 [18]. Geopolitical conflicts, climate change, and economic instability have compounded food insecurity, disrupted supply chains and exacerbated malnutrition among vulnerable populations [1, 51]. Addressing these challenges requires strengthening food systems, promoting sustainable agriculture and investing in climate‐resilient practices [18]. This burden is tightly coupled with climate change and conflict, and the bidirectional links between food insecurity, climate shocks and undernutrition are analysed in Section 12 [13].

## 8 Health Workforce Shortages

The global health workforce crisis continues to deepen in 2025, with the WHO projecting a shortfall of 11 million health workers by 2030, mostly in low‐ and lower‐middle‐income countries [20]. The WHO African Region faces a projected shortage of 6.1 million health workers by 2030, which sustains regional disparities in healthcare delivery [52]. Budget cuts and economic constraints in many countries are expected to reduce the capacity to absorb new health workers, thereby worsening existing shortages [53].

Addressing the health workforce crisis requires the expansion of education and training programmes, improvement of working conditions and compensation, reduction of the migration of health workers from low‐ to high‐income settings and use of digital health technologies to extend the reach of existing workers [20, 54]. Investment in community health workers and task‐shifting strategies can help bridge the gaps in service delivery, particularly in rural and underserved areas [53]. Because an adequately staffed workforce underpins progress on every other challenge in this perspective, workforce investment functions as a cross‐cutting enabler in the proposed agenda in Section 12 [20]. Short‐term measures include ethical management of international recruitment under the WHO Global Code of Practice and rapid scale‐up of community health workers, whereas long‐term resilience depends on domestic financing for training and retention rather than reliance on inward migration [19, 55].

## 9 Substance Use and Substance Use Disorders

Behavioural risks compound these system‐level constraints. Substance use and substance use disorders remain major global public health concerns in 2025. Alcohol use alone causes approximately 2.6 million deaths annually (based on 2019 data), accounting for 4.7% of all global deaths, whereas an additional 0.6 million deaths are attributable to psychoactive drug use [22]. The 2025 World Drug Report from the United Nations Office on Drugs and Crime revealed that 316 million people aged 15–64 used drugs in 2023, excluding alcohol and tobacco, a 28% increase over the past decade that outpaced global population growth [21].

Global cocaine production reached a record 3708 tonnes in 2023, up one‐third from 2022, whereas the number of cocaine users grew from 17 million in 2013 to 25 million in 2023 [21]. The treatment gap is severe, with only 1 in 12 people with drug use disorders estimated to have received any form of treatment in 2023 [21]. An estimated 400 million people live with alcohol use disorders globally, of whom 209 million live with alcohol dependence [22].

Global tobacco use has declined substantially, dropping from 33% of adults in 2000 to below 20% by 2024; however, this progress is challenged by the rapid rise of underregulated products such as vaping devices, non‐combustible tobacco and nicotine pouches [56], coupled with youth‐targeted marketing. For instance, the swift growth of nicotine pouch use among teenagers and young adults signals a transformation in nicotine consumption, with serious implications for public health.

Marketed as discreet and ‘tobacco free’, these products are perceived as safer than smoking; however, their cardiovascular risks have not been extensively explored. Key concerns include high nicotine content, efficient bloodstream delivery and increasing dual or poly use with other nicotine products. Adolescents are especially vulnerable given their continuing neurological development and higher addiction risk [23]. Effective public health strategies must include stricter regulations on substance availability, expanded access to evidence‐based treatment and recovery services, and community‐based prevention programmes that address the underlying social and economic determinants of substance use [22]. Another emerging concern is the spread of highly potent novel synthetic opioids, particularly nitazenes, in the unregulated drug supply of several high‐income settings, which sharply increases the risk of overdose and complicates surveillance and treatment [57]. However, the coverage of effective responses remains low, since a global systematic review found that only about 18 of every 100 people who inject drugs access opioid; therefore, closing the treatment gap will require scaling opioid agonist therapy, naloxone and community‐based harm reduction and integrating substance‐use care into primary health services, particularly in LMICs [58].

## 10 Sexual and Reproductive Health Equity

Access to care is shaped as much by rights and gender as by supply. Ensuring equitable access to sexual and reproductive health and rights remains a persistent global challenge in 2025. In 2023, an estimated 260,000 maternal deaths occurred worldwide, representing 712 fatalities per day. This represents a 40% decline in the maternal mortality ratio since 2000 [59]. However, an estimated 164 million women of reproductive age have unmet needs for contraception [60]. Contraceptive use alone prevented an estimated 77,400 maternal deaths in 2023, representing approximately 24% of maternal deaths that year [25].

Gender‐based violence is intrinsically linked to sexual and reproductive health, affecting one in three women in their lifetime and constraining their ability to exercise their sexual and reproductive rights [24, 29]. Limitations on reproductive choice, including restricted access to safe abortion services, violate women's bodily autonomy and their right to make informed decisions regarding sex and childbearing [61]. A 2025 report by the Organisation for Economic Co‐operation and Development emphasizes the urgent need to transform laws and norms to achieve universal sexual and reproductive health and rights [62].

Ending early marriage and adolescent pregnancy remains a significant challenge. Each year, approximately 21 million girls aged 15–19 years become pregnant in LMICs [26]. Approximately half of these pregnancies are unintended (2019 estimates) [63]. Adolescent pregnancy is associated with increased risks of death for both the mother and child, a higher likelihood of mental health issues, and limitations on educational and economic opportunities, which, in turn, result in intergenerational cycles of poverty and inequality [26]. Achieving sexual and reproductive health equity requires integrating these services into universal health coverage, addressing gender‐based disparities through legal reforms and community education, and prioritizing investment in comprehensive sexuality education and family planning services [64]. A pressing 2025‐specific challenge is the contraction of US foreign assistance for reproductive health, as recent reductions in donor funding threaten contraceptive supplies and maternal health services in the most aid‐dependent settings, with a modelling study projecting large increases in unintended pregnancies and unsafe abortions if the shortfall is not replaced [65]. In parallel, self‐managed medication abortion and telemedicine‐supported self‐care are widening access in restrictive and low‐resource contexts, although realizing their benefits safely depends on supportive regulations, quality assurance and follow‐up monitoring [66].

## 11 AI, Digital Health and Public Health Ethics

The final challenge concerns not the disease but the tools increasingly used against it. The rapid integration of AI and digital technologies into healthcare presents both opportunities and ethical challenges for public health in 2025. The WHO has published updated guidance on the ethics and governance of AI for health, addressing large multimodal models and their growing applications in diagnosis, treatment, health research, drug development and public health surveillance [27]. The Global Initiative on AI for Health serves to harmonize governance standards and advance the ethical, regulatory and operational dimensions of health‐related AI, with particular attention to LMICs [28].

A 2025 WHO assessment found that AI is reshaping health systems across the European Region, revealing varied levels of readiness and the need for regulatory frameworks to ensure safe and effective deployment [67]. Key ethical challenges include addressing biases in healthcare data based on race, ethnicity, age and gender that are encoded in algorithms, ensuring data security and privacy in digital health technologies, and eliminating the pre‐existing digital divide that limits access to these tools in LMICs and among marginalized populations in wealthier nations [68]. A defining 2025 trend is the rapid clinical uptake of generative AI and large language models for tasks such as clinical documentation, triage and diagnostic support, which is outpacing prospective evaluation, as a systematic review of healthcare applications found that only 5% of 519 studies used real patient‐care data [69]. Persistent implementation challenges include the scarcity of prospective clinical validation and post‐deployment monitoring, the risk of algorithmic bias, and fragmented data governance, which highlights the need for risk‐based regulation and enforced safety standards for health AI [70]. Realizing the benefits of AI in health while limiting its risks depends on transparency, accountability, and human oversight at every stage of design, deployment, and use [27], and on regulations that keep pace with clinical adoption [28].

## 12 Cross‐Cutting Interconnections and a Proposed Agenda for Action (2025–2030)

The 10 challenges identified in this perspective are not isolated silos but a syndemic constellation in which biological, social and ecological pathways interact and amplify each other [39, 71]. Climate change drives food insecurity through droughts and heatwaves, which, in turn, worsens malnutrition, infectious disease susceptibility and the burden of NCDs through pathways such as anaemia and micronutrient deficiency [13]. AMR and climate change are interlinked through accelerating pathogen spread, antibiotic overuse in disrupted health systems and One Health vulnerabilities at the animal–environment–human interface [4]. The mental health burden is compounded by climate‐related displacement, gender‐based violence, food insecurity, conflict and social isolation, which intensified during and after the COVID‐19 pandemic [5]. Health workforce shortages constrain progress across every other challenge area, since neither the 2025 WHO Pandemic Agreement nor universal health coverage commitments can be operationalized without an adequately trained and supported workforce [20]. The rapid integration of AI in health threatens to widen the digital divide between high‐ and LMICs if not paired with equitable access and rights‐based governance [28]. Recognizing these bidirectional interdependencies reinforces the case for One Health, intersectoral collaboration and integrated programming as the foundational architecture of the post‐2025 global health agenda [72].

Translating this interconnected analysis into action requires a coherent agenda for 2025–2030 that includes building on the Sustainable Development Goals, the WHO Fourteenth General Programme of Work [73], the 2025 WHO Pandemic Agreement [17], the 2024 United Nations General Assembly Political Declaration on AMR [31], and the climate and health declarations under the United Nations Framework Convention on Climate Change [34]. Table 2 maps each of the 10 challenges to (a) priority actions for the next 5 years, (b) the main responsible actors, (c) feasible implementation mechanisms, (d) suggested monitoring indicators and (e) specific implications for LMICs and equity considerations, respectively. The actions in Table 2 were selected by consensus as evidence‐based, feasible within the current global health architecture, and equity‐sensitive. They were not scored or formally ranked against one another, and the rows follow the order of the challenge sections rather than any order of priority; the table is therefore described as a proposed rather than prioritized agenda. Progress across the 10 challenges requires both vertical strengthening of disease‐specific programs and horizontal integration across health systems.

**TABLE 2:** Proposed action agenda for the Top 10 public health challenges, 2025–2030.

Challenge	Priority actions (2025–2030)	Main responsible actors	Implementation mechanisms	Monitoring indicators	LMIC and equity considerations
Antimicrobial resistance	Scale AWaRe stewardship, accelerate R&D, and strengthen One Health surveillance	WHO–FAO–UNEP–WOAH Quadripartite; National Drug Regulators	National One Health Action Plans, pooled R&D financing and GLASS	Resistance rates; antibiotic consumption per 1000 population	LMIC laboratory capacity and access to new antimicrobials
Climate change and health	Integrating health into NDCs, building climate‐resilient facilities and expanding heat early warning systems are essential	WHO, UNFCCC, ministries of health and environment and climate funds	Health Adaptation Plans and COP commitments	Heat‐related mortality, vector‐borne disease incidence and climate finance for health	Adaptation finance for LMICs: protect outdoor and informal workers
Mental health burden and treatment gaps	Integrating mental health into primary care, scaling community services and raising budget shares	WHO; ministries of health, education and social welfare; and employers	WHO Special Initiative for Mental Health, mhGAP and UHC benefit packages	Mental health budget share, treatment coverage and suicide mortality	Task‐shifting in LMICs: gender‐ and trauma‐informed care
Noncommunicable diseases and health disparities	Implement the WHO NCD Best Buys and strengthen tobacco, alcohol, sugar and salt regulation	WHO; ministries of health and finance; food and beverage regulators	WHO Global Action Plan for NCDs; UN High‐Level Meeting on NCDs	Premature NCD mortality (30–70 years) and obesity and hypertension prevalence	Affordable insulin and cancer care in LMICs: commercial determinants
Pandemic preparedness and infectious disease threats	Operationalize the Pandemic Agreement and 2024 IHR amendments and finance the Pandemic Fund	WHO, governments, the Pandemic Fund, Gavi and CEPI	PABS system, WHO R&D Blueprint, SPAR and JEE reporting	Childhood immunization coverage, SPAR and JEE scores	Technology transfer to LMICs: immunization in fragile settings
Food insecurity and malnutrition	Scale climate‐resilient agriculture, expand social protection and integrate nutrition into primary care	FAO; WFP; IFAD; UNICEF; ministries of agriculture	SDG 2: Food system transformation pathways	Undernourishment, food insecurity, food affordability and child stunting	Smallholder and women farmer support; affordable healthy diets
Health workforce shortages	Expanding education and training, improving retention and pay, and ethically governing migration are essential	WHO, ministries of health and education and World Bank	WHO Global Code of Practice; National Health Workforce Account	Health worker density per 1000; skill mix; migration outflows	Africa Health Workforce Investment Charter, rural deployment incentives
Substance use and substance use disorders	Strengthen tobacco, alcohol and drug policies; expand treatment and harm reduction	WHO, UNODC, governments, customs and tax authorities	WHO FCTC; SAFER framework; UNGASS commitments	Tobacco, alcohol and drug use prevalence; treatment coverage	Affordable opioid agonist therapy: Protecting youth from marketing
Sexual and reproductive health equity	Integrate SRH into UHC benefit packages, expand contraceptive choice and address gender‐based violence	WHO, UNFPA, UN Women and the ministries of health and justice	SDG 3.7 and 5.3; UNFPA Strategic Plan	Maternal mortality ratio, contraceptive prevalence and adolescent birth rate	Adolescent and humanitarian SRH services; rights in conflict settings
Artificial intelligence, digital health and public health ethics	Implement WHO AI ethics guidance and strengthen data protection and algorithmic accountability	WHO, GI‐AI4H and national data protection regulators	WHO Guidance on Ethics and Governance of AI for Health; National AI Strategies	AI uptake in LMIC health systems; algorithmic bias audit coverage	Closing the digital divide, LMIC data sovereignty and inclusive training data

Abbreviations: AWaRe, Access, Watch and Reserve antibiotic classification; CEPI, Coalition for Epidemic Preparedness Innovations; COP, Conference of the Parties; FCTC, Framework Convention on Tobacco Control; GI‐AI4H, Global Initiative on Artificial Intelligence for Health; GLASS, Global Antimicrobial Resistance and Use Surveillance System; IHR, International Health Regulations; JEE, Joint External Evaluation; LMIC, low‐ and middle‐income country; mhGAP, Mental Health Gap Action Programme; NDCs, nationally determined contributions; PABS, pathogen access and benefit‐sharing; R&D, research and development; SAFER, WHO alcohol control initiative; SDG, Sustainable Development Goal; SPAR, State Party Self‐Assessment Annual Reporting; SRH, sexual and reproductive health; UHC, universal health coverage; UNGASS, United Nations General Assembly Special Session; WOAH, World Organisation for Animal Health.

The effective implementation of this agenda depends on three enabling conditions. First, financing must shift from emergency response cycles to predictable, long‐term investments in primary healthcare, the health workforce and surveillance infrastructure. Second, equity must be the primary lens for prioritization, with explicit targeting of populations bearing disproportionate burdens, such as women, children, indigenous communities, refugees, migrants and people in conflict‐affected settings. Third, accountability mechanisms must be transparent and grounded in standardized monitoring indicators that allow countries and global bodies to track progress in real time. These enabling conditions, combined with the proposed actions in Table 2, offer a pragmatic pathway from descriptive analysis to coordinated, equity‐centred action across the post‐2025 global health agenda. A practical sequencing underpins this agenda, distinguishing short‐term actions achievable within the existing architecture, such as regulatory enforcement, stewardship and closing immunization and treatment gaps, from long‐term structural investments in the workforce, surveillance and research and development that require sustained multi‐year financing.

However, this perspective has several limitations. The identification of the 10 challenges rests on structured expert judgement by the author team rather than on a systematic or scoping review, and a different group applying the same criteria might have arrived at a different set. The indicators presented are drawn from different sources, reference years and methodologies and are, therefore, not always directly comparable with one another. Global estimates conceal substantial regional, national and subnational variations, and several estimates for 2025 are modelled values that may be revised as further data become available. Finally, the actions proposed in Table 2 are generic and must be adapted to national legal frameworks, financing, political conditions and health system capacity before implementation.

## 13 Conclusion

Four messages emerged from this assessment. First, the 10 challenges are interdependent rather than separate; therefore, integrated and One Health‐informed programming is more efficient than parallel vertical responses. Second, equity determines outcomes: LMICs and marginalized populations within all countries carry a disproportionate share of the burden, and interventions that are not explicitly targeted will widen the existing gaps. Third, the health workforce is the binding constraint because neither the WHO Pandemic Agreement nor universal health coverage commitments can be delivered without an adequate and well‐supported workforce. Fourth, the governance frameworks agreed upon in 2025 create an opportunity that will be realized only if they are matched by predictable financing, completion of outstanding legal and implementation steps and transparent accountability against standardized indicators. The period until 2030 will determine whether the commitments made in 2025 translate into measurable improvements in health.

## Author Contributions

Don Eliseo Lucero Prisno III conceptualized and designed the study. Zhinya Kawa Othman, Olalekan John Okesanya and Shuaibu Saidu Musa performed the literature review and data curation. Mohamed Mustaf Ahmed and Uthman Okikiola Adebayo drafted the manuscript. Francesco Branda, Jerico Bautista Ogaya, Rahmatullah Nazari and Abdullahi Hassan Elmi revised the manuscript. Don Eliseo Lucero Prisno III supervised the study. All authors have read and approved the final manuscript.

## Funding

The authors have nothing to report.

## Ethics Statement

The authors have nothing to report.

## Conflicts of Interest

The authors declare no conflicts of interest.

## Data Availability

All data discussed in this perspective are drawn from publicly available institutional reports and peer‐reviewed publications; the original sources are cited inline in the text as hyperlinked DOIs or URLs. No new primary data were created or analysed.
